# GTW inhibits the Epithelial to Mesenchymal Transition of Epithelial Ovarian Cancer via ILK/AKT/GSK3β/Slug Signalling Pathway

**DOI:** 10.7150/jca.52418

**Published:** 2021-01-01

**Authors:** Ying Feng, Fuyin Le, Puyuan Tian, Yanying Zhong, Fuliang Zhan, Genhua Huang, Hui Hu, Tingtao Chen, Buzhen Tan

**Affiliations:** 1 Department of Obstetrics & Gynecology, The Second Affiliated Hospital of Nanchang University, Nanchang, Jiangxi 330006, PR China.; 2 Institute of Translational Medicine, Nanchang University, Nanchang, Jiangxi 330031, PR China.

**Keywords:** epithelial ovarian cancer (EOC), epithelial-mesenchymal transition (EMT), A2780/DDP cells, Cisplatin (DDP) resistance, *Tripterygium* glycosides (GTW), ILK/AKT/GSK3β/Slug pathway

## Abstract

**Background:** Epithelial ovarian cancer (EOC) accounts for the most lethal of all gynaecological cancers which is attributed to metastasis, invasiveness and drug resistance. A crucial link has been found between epithelial-mesenchymal transition (EMT) and cancer metastasis and chemo-resistance. Previous studies have confirmed that one of the main components of *tripterygium* glycosides (GTW)-triptolide (TPL) has anticancer effects.

**Methods:** The purpose of this study is to determine whether GTW could inhibit EMT in A2780/DPP cells *in vitro* and *in vivo*, and explore the underlying mechanism.

**Results:** *In vitro* results showed that GTW inhibited cell proliferation, invasion and migration, and intensified the sensitivity of A2780/DDP cells to cisplatin (DDP). GTW, especially GTW+DDP, significantly inhibited the expression of N-cadherin, integrin-linked kinase (ILK), phospho-protein kinase B/AKT (PKB/p-AKT), phospho-glycogen synthase kinase (p-GSK3β) and Slug, while it increased E-cadherin levels by inhibiting EMT via the ILK/AKT/GSK3β/Slug signalling pathway. Animal results indicated that GTW, especially GTW+DDP, significantly reduced tumour burden, prolonged the life span of mice, and down-regulated the levels of tumour markers CA125 and HE4 by regulating EMT through the ILK/AKT/GSK3β/Slug signalling pathway.

**Conclusion:** Our results highlighted the significance of EMT in EOC metastasis, invasiveness and resistance to DDP and investigated the potential role of GTW as an adjuvant therapeutic agent in chemo-resistant EOC.

## Introduction

Ovarian cancer is the deadliest gynaecological malignancy and the main cause of cancer-related deaths in women, with epithelial ovarian cancer (EOC) being the most common type of malignant ovarian tumour, occurring in 90% of cases 1. The treatment of ovarian cancer broadly encompasses surgical treatment, radiotherapy and chemotherapy, molecular targeted therapy, immunotherapy and photodynamic therapy, depending on cancer stage, the histopathological type, the age of the patient, the desire of the patient to have children and general health 2, 3. EOC patients often presents with advanced stage, often received cytoreductive surgery followed by platinum-based chemotherapy, and 80% of these women will develop platinum-resistant and relapse within 12-18 months of their primary treatment 4, 5. Therefore, there is an urgent need to develop new therapies to improve patient prognosis.

Drug resistance is a multi-factorial phenomenon involving various mechanisms, characterised by decreasing cisplatin uptake, enhancing DNA damage tolerance and repair, increasing the efflux of cisplatin caused by cellular glutathione or metallothionein cause 6-8. Moreover, recent reports have indicated that cancer progression, migration, invasion, and chemotherapeutic resistance may occur as a result of epithelial-mesenchymal transition (EMT) 9. The acquisition of an EMT phenotype has been demonstrated in paclitaxel-resistant ovarian cancer cells 10, tamoxifen/adriamycin-resistant breast cancer cells 11, gemcitabine/5-FU-resistant pancreatic cancer cells 12, gefitinib-resistant non-small cell lung cancer and oxaliplatin-resistant colorectal cancer cells 13, and blockade of the EMT pathways is critical for preventing cancer cell migration and invasion as well as for restoring drug sensitivity 14, 15.

Several key signalling pathways, including the phosphatidylinositol 3-kinase (PI3K) pathway, are known to be involved in EMT 16. As a component of the PI3K pathway located upstream of protein kinase B/AKT (PKB/AKT), integrin-linked kinase (ILK) is a serine/threonine protein kinase, which was originally identified by interacting with β1 and β3 integrin ligands 17. ILK has been shown to play a fundamental role in regulating the survival, proliferation, migration, invasion, and angiogenesis of cells by mediating the integrin signal in various cells 18-20. It also plays an important role in EMT for activated ILK via phosphorylating its downstream target kinases, PKB/AKT at Ser 473 and glycogen synthase kinase (GSK3β) at Ser 9 21, which in turn affects cell survival, cell cycle, and changes extracellular matrix (ECM) modifications and cell adhesion 22. Thus, EOC patients may benefit from targeted therapies that inhibit EMT.

*Tripterygium* glycosides (GTW) is a fat-soluble mixture obtained from the roots of the genus *Tripterygium wilfordii*. It has been approved by the China State Food and Drug Administration (Z32021007) for the conventional treatment of inflammatory bowel disease (IBD) (Crohn's disease (CD) and ulcerative colitis (UC)) 23, 25 and rheumatoid arthritis (RA) 24. In addition GTW possess other biological activities for the treatment of lupus, cancer, and nephritic syndrome 26, 27. Recent studies have shown that triptolide (TPL), one of the main components of GTW, could inhibit EMT through the PI3K/AKT/mTOR pathway to exert anti-tumour effect 28. In the previous research by our team, we verified the effect of TPL on drug-resistant EOC cells *in vitro* and* in vivo* 29-31. Our group indicated that TPL had synergistically enhanced the cytotoxicity of cisplatin (DDP), and promoted the apoptosis of the SKOV3^PT^ cells through the inhibition of nuclear factor kappa-B (NF-κB) in a p53-independent pathway inducing mitochondria-derived reactive oxygen species (ROS) accumulation 29. However, whether GTW has an inhibitory effect like TPL on the proliferation, invasion and metastasis of drug-resistant EOC remains unclear.

Our study aimed to investigate whether GTW could inhibit metastasis and enhance the sensitivity of drug-resistant human EOC cells (A2780/DDP) to DDP by suppressing EMT via targeting the ILK/AKT/GSK3β/Slug signalling pathway, and explore the underlying mechanism.

## Materials and methods

### Cell lines and culture

The cisplatin-resistant A2780/DDP cell line (human epithelial ovarian carcinoma-derived) was purchased from Cell Bank of Chinese Academy of Sciences (Shangha, China). A2780/DDP was cultured in RPMI-1640 medium (Gibco Life Technologies, Grand Island, NY, USA) containing 10% foetal bovine serum (Biological Industries, Israel), 100 U/mL penicillin/streptomycin (Solarbio, China) in a 37 °C incubator containing 5% CO_2_.

### Cell viability assay

A2780/DDP cells at logarithmic growth stage were seeded in 96-well plates with 1000 cells/well with five replicates for each test condition, and allowed to attach overnight in a humidified atmosphere containing 5% CO_2_ at 37 °C. Then, the cells were exposed to 100 µL/well various concentrations of GTW (Preferred, Chengdu, China) (0, 50, 200, 800, 1600, 3200 µg/mL) and DDP (Hansoh Pharma, China) (0.5625, 1.25, 2.5, 5, 10 and 20 µg/mL) for 24 h. After incubation, 20 µL of fresh medium containing 10 µL of CCK-8 solution (Beijing Zoman Biothchnology Co.,Ltd, China) was added to each well and incubated for 1.5 h at 37 °C. The absorbance at 450 nm was measured by a micoplate reader (Bio-Rad, CA, USA).

### Cellular migration and invasion assays

A 24-well Boyden chamber (8 μm pore size; Corning Costar, USA) was used for transwell migration assay. Here, 2 × 10^4^ or 4 × 10^4^ A2780/DDP cells were loaded onto the top of the 24-well migration chamber in 100 µL serum-free media, and filled 600 µL medium containing 20% FBS into the lower chamber. Cells after incubating with a range concentrations of GTW (0, 50, 200, 800, 1600 and 3200 µg/mL) and a various concentrations of DDP (0.5625, 1.25, 2.5, 5, 10 and 20 µg/mL) in the incubator for 24 h, the cells has migrated into the lower surface of the filter were fixed with 4% paraformaldehyde for 15 minutes, and then stained with 0.1% crystal violet solution for 30 minutes. Pictures (100×) were taken with an Olympus IX51 (Olympus Optical, Melville, NY, USA) inverted microscope and 5 visual fields were counted. The sterile Borden chamber was also used for invasion measurements. BD Matrigel (BD Biosciences, USA) was placed in a -4 °C refrigerator overnight before commencing the experiment, at which point the BD matrix will become liquid. It was diluted with serum-free medium in an 1:8 ratio. When the upper chamber has been pre-coated with Matrigel, operations similar to migration analysis were performed.

### Small interfering RNA (siRNA) transfection

Slug (U) and ILK (I) specific siRNA were purchased from RIBO Company (Shanghai, China). The logarithmic growth phase A2780/DDP cells were divided into 8 groups: (1) blank control group; (2) DDP group (10 µg/mL DDP for 24 h); (3) GTW group (800 µg/mL GTW for 24 h); (4) DDP+GTW group (800 µg/mL GTW and 10 µg/mL DDP for 24 h); (5) U or I (10 μM for 24 h); (6) DDP+U or DDP+I group; (7) GTW+U or GTW+I group; and (8) DDP+GTW+U or DDP+GTW+I group. For siRNA transfection, A2780/DDP cells were planted in 6-well plates without streptomycin treated with 50-60% confluence before transfection. Cell transfection was carried out using GeneTran III Transfection Reagent (Biomiga, Inc., USA). After incubation for 6 h, the culture medium was changed into fresh RPMI-1640 medium containing 10% FBS. After 48 h of transfection, cells were harvested for the following cell experiments. For A2780/DDP the sequence for Slug specific siRNA is 5'-CCCAUUCUGAUGUAAAGAATT-3', 5'-UUCUUUACAUCAGAAUGGGTT-3', respectively. Also, the sequence for ILK specific siRNA is 5'-GACCCAAAUUUGACAUGAUTT-3', 5'-AUCAUGUCAAAUUUGGGUCTT-3', respectively, in A2780/DDP cells.

### Detection of AKT and GSK3β activity using their inhibitor

The AKT inhibitor (A) and GSK3β inhibitor (S) were purchased from APExBIO (Houston, USA). The logarithmic growth phase A2780/DDP cells were divided into 8 groups: (1) blank control group; (2) DDP group (10 µg/mL DDP for 24 h); (3) GTW group (800 µg/mL GTW for 24 h); (4) DDP+GTW group (800 µg/mL GTW and 10 µg/mL DDP for 24 h); (5) A or S (10 µM for 24 h); (6) DDP+A or DDP+S group; (7) GTW+A or GTW+S group; and (8) DDP+GTW+A or DDP+GTW+S group. A2780/DDP cells (5×10^5^ per well) were plated in six-well plates overnight and the experimental conditions were set in triplicate. Then MK2206 (AKT inhibitor, 10 μM) or AR-A014418 (GSK3β inhibitor, 10 μM) was added to prevent AKT or GSK3β activity. After 24 h of incubation, cells were collected for subsequent experiments.

### Western blotting

The protein of cells were lysed in RIPA lysis buffer (Solarbio, China) on ice, and the tumour tissues were homogenised in RIPA lysis buffer (Solarbio, China), before samples were centrifuged at 10000 rpm for 30 min at 4 °C. The protein concentration of the samples was measured using BCA kit (Thermo scientific, USA), subsequently, they were boiled for 5-10 min in 1× protein loading buffer. The proteins were resolved using polyacrylamide gel electrophoresis, electrotransferred to the polyvinylidene difluoride membranes, and then nonspecific binding sites were blocked using 5% bovine serum albumin (BSA) (Servicebio, China) in Tris buffered saline with Tween 20 (TBST) (Solarbio, China) for 2 h at room temperature. Polyvinylidene fluoride (PVDF) membranes were then incubated with the following primary antibodies at 4 °C overnight: anti-β-actin (1:5000; Proteintech, Cat# 60008-1-lg; RRID: not registered; having reactivity with mouse), anti-AKT (1:1000; Proteintech, Cat# 10176-2-AP; RRID: AB_2224574; having reactivity with mouse), anti-p-AKT (1:2000; Proteintech, Cat# 66444-1-lg; RRID: not registered; having reactivity with mouse), anti-GSK3β (1:1000; Proteintech, Cat# 22104-1-AP; RRID: not registered; having reactivity with mouse), anti-p-GSK3β (1:1000; Proteintech, Cat# 14850-1-AP; RRID: not registered; having reactivity with mouse), anti-ILK (1:1000; Proteintech, Cat# 12955-1-AP; RRID: AB_2127053; having reactivity with mouse), anti-Slug (1:1000; abcam, Cat# ab180714; RRID: AB_2728773; having reactivity with mouse), anti-N-cadherin (1:2000; Proteintech, Cat# 22018-1-AP; RRID: AB_2813891; having reactivity with mouse), and anti-E-cadherin (1:5000; Proteintech, Cat# 20874-1-AP; RRID: AB_10697811; having reactivity with mouse). Cells were washed with TBST buffer, and then appropriate amounts of secondary antibody, e.g. goat anti-rabbit secondary antibody (1:1000; Abcam, Cat# 205718; RRID: not registered), or goat anti-mouse secondary antibody (1:1000; Abcam, Cat# 97265; RRID: not registered), conjugated with horseradish peroxidase (HRP) was added for another 1.5 h at room temperature.

### Mouse model of ovarian cancer

Female nude mice, aged 6 weeks, were purchased from Hunan SJA Laboratory Animal Co., Ltd. (Changsha, Hunan, China; RRID: MGI_5656552) randomly divided into four groups (N = 12 per group), and acclimatised for one week. Mice received 1×10^6^ A2780/DDP cells subcutaneously suspended in PBS. When the tumour reaches 50 mm^3^, mice were treated as follows: (1) Model (50 mL/kg/day PBS was intraperitoneally injected for a total of 14 days); (2) DDP (4 mg/kg/day DPP was intraperitoneally injected for the first and eighth day, 2 days in total); and (3) GTW (0.15 mg/kg/day GTW gavage, 0.15 mg/kg/day for a total of 14 days), DDP + GTW (4 mg/kg/day DPP was intraperitoneally injected for the first and eighth day, 2 days in total and 0.15 mg/kg/day GTW gavage, 0.15 mg/kg/day for a total of 14 days). The tumour volume was measured twice a week and weighed the mice. At day 22, three mice from each group were euthanized; their tumours were removed and frozen at -80°C for further study. The remaining mice were used to assess survival curves. Tumour volume was determined as follows:

Tumour volume = length × width^2^ × 0.5 (repeated three times)

### ELISA

Blood was collected from mice of each group (day 22) via retro-orbital bleeding. Fresh blood was allowed to stand at room temperature for 10 minutes, and then spun in a centrifuge (3000 rpm, 10 min). After centrifugation, the serum fraction was immediately transferred to a new centrifuge tube and stored at -80 °C until use. The levels of tumour markers (CA125 and HE4) in serum samples were determined through the mouse ELISA kits (both from eBioscience, USA).

### Statistical analysis

Statistical analysis was performed by one-way ANOVA or two-way ANOVA and expressed as mean ± SD. *P* < 0.05 indicates a statistically significant difference.

## Results

### GTW prohibits the proliferation, migration and invasion characteristics of A2780/DDP cells

**Figure 1:**
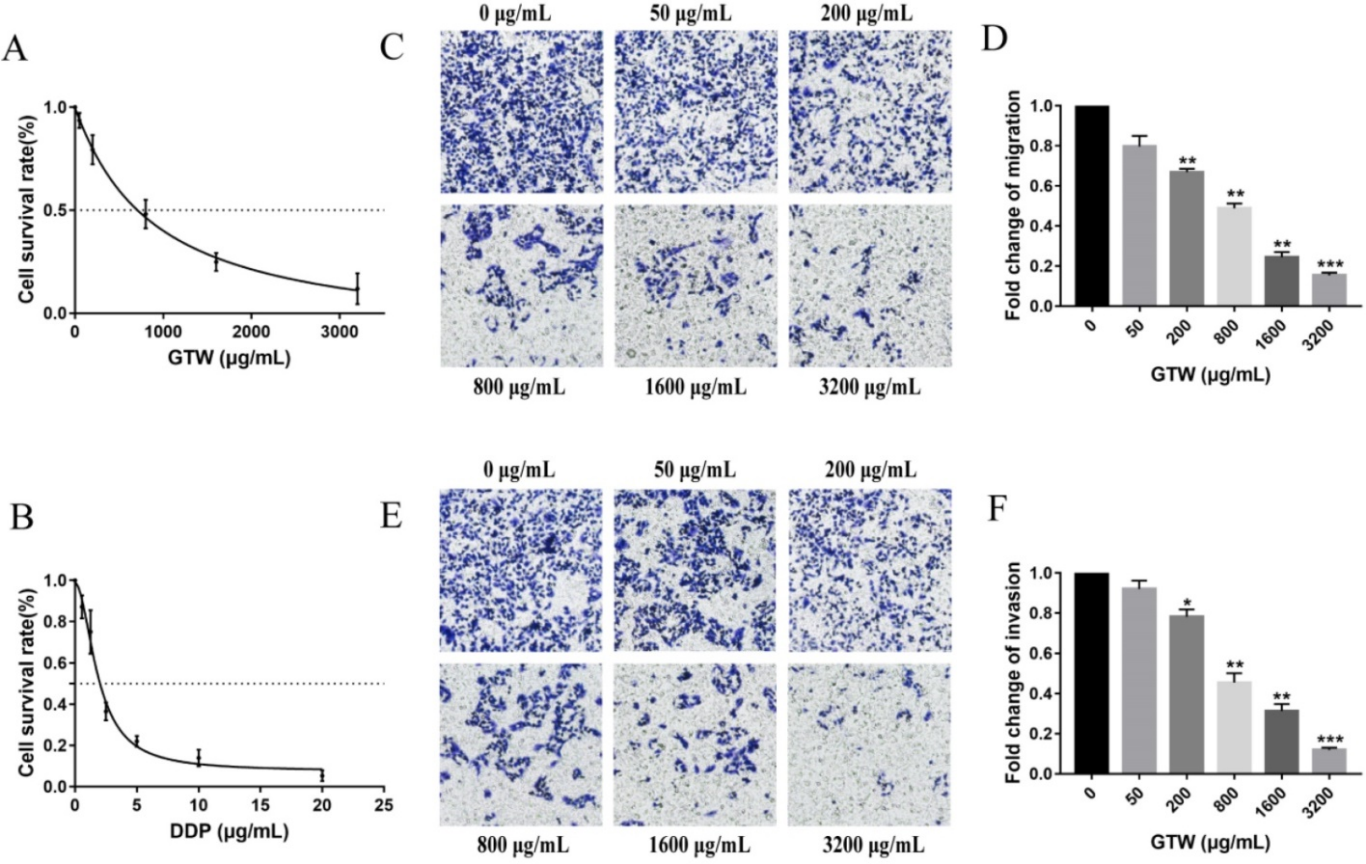
GTW inhibited the proliferation, migration, and invasion capabilities of A2780/DDP cells *in vitro*. (A) A2780/DDP cells were treated with a range of concentrations of GTW for 24 hours to examine cell survival; (B) A2780/DDP cells were treated with a range of concentrations of DDP for 24 hours to examine cell survival; (C-F) Transwell migration and invasion assay of A2780/DDP cells after treatment with increased concentrations of GTW (×100 magnification). ∗ *P* < 0.05, ∗∗ *P* < 0.005, and ∗∗∗ *P* < 0.001.

To detect the effect of GTW on the proliferation of epithelial drug-resistant EOC cells, A2780/DDP cells were treated with a range of concentrations of GTW (0, 50, 200, 800, 1,600 and 3,200 µg/mL) for 24 hours. As shown in Figure 1A, GTW had the effect of inhibiting the proliferation of A2780/DDP cells in a dose-dependent manner, and the IC_50_ of GTW on A2780/DDP cells was 882.1 µg/mL. Furthermore, we treated A2780/DDP cells with DDP (0.5625, 1.25, 2.5, 5, 10 and 20 µg/mL) (Figure 1B); similar results were obtained showing that the cell proliferation inhibition of DDP was dose-dependent, as the IC_50_ of DDP on A2780/DDP cells was 1.891 µg/mL. Transwell migration and invasion tests were used to determine the influence of GTW on the migration and invasion abilities of A2780/DDP cells. As expected, the migration and invasion abilities of A2780/DDP cells were significantly inhibited after 24 hours of GTW treatment (Figure 1C-F) (*P* < 0.05). All of the results showed that GTW had a significant inhibitory effect on proliferation, migration and invasion of A2780/DDP cells *in vitro.*

### GTW inhibited EMT in A2780/DDP cells by targeting the Slug

**Figure 2:**
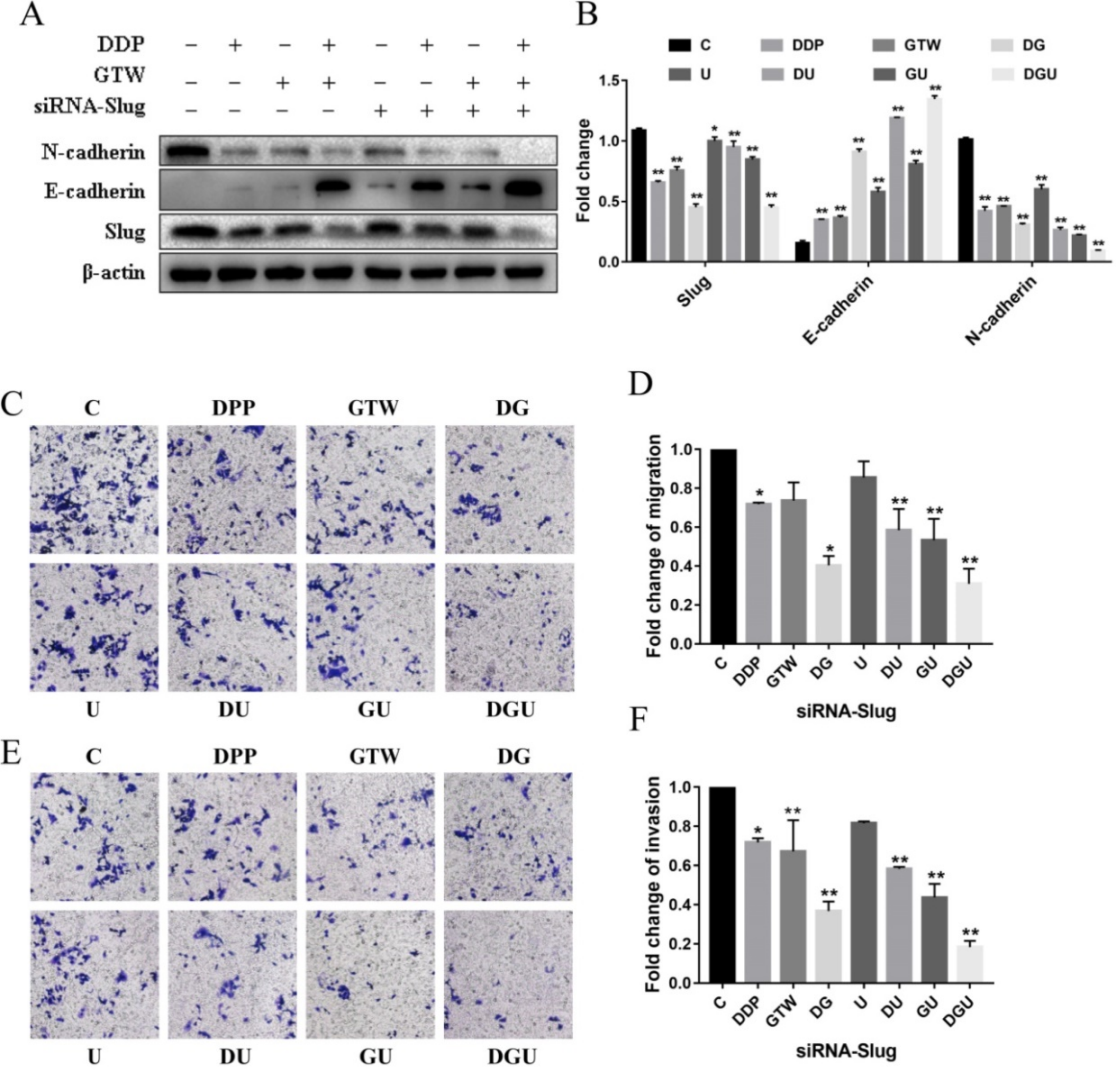
GTW inhibited EMT in A2780/DDP cells through targeting Slug. (A-B) A2780/DDP cells were transfected with siRNA-Slug and cultured for an additional 48 h, then incubated with GTW (800 µg/mL) or DDP (10 µg/mL) for 24 hours. Western blot analysis for the expression of Slug, E-cadherin, and N-cadherin protein; (C-F) Transwell migration and invasion assay of A2780/DDP cells after transfection with siRNA-Slug combined with GTW, DDP or GTW+DDP treatment. *P < 0.005, **P < 0.001.

In view of the fact that GTW can effectively suppress the migration and invasion of A2780/DDP cells, we used western blot to detect the expression of EMT-related factors to assess whether GTW could affect EMT and sensitise the drug sensitivity of drug-resistant of EOC cells to DDP. As shown in Figure 2A-B, supplementation with GTW or DDP up-regulated the expression of epithelial marker E-cadherin and down-regulated the mesenchymal marker N-cadherin to a certain extent compared with the control group (*P* < 0.005). In addition, the expression of the transcription factor Slug was also inhibited after exposure to GTW or DDP. Furthermore, GTW combined with DDP played a synergistic effect on the up- and down-regulation of the above proteins (*P* < 0.005). To confirm the role of Slug channel in EMT, specific siRNA was performed to interfere with the production of Slug in the A2780/DDP cell line. After transfection of siRNA-Slug, the expression of Slug and N-cadherin was reduced while the expression of E-cadherin was increased in A2780/DDP cells, and siRNA-Slug intensified the synergistic effect of GTW and DDP combination (*P* < 0.005). Additionally, we also studied the effects of Slug silencing on cell migration and invasion followed by GTW, DDP and GTW+DDP treatment (Figure 2C-F). Unsurprisingly, the migration and invasion abilities of cells were suppressed most effectively in siRNA-Slug treatment followed by treatment with the combination of GTW+DDP (*P* < 0.005).

### GTW inhibited EMT by targeting ILK in A2780/DDP cells

**Figure 3:**
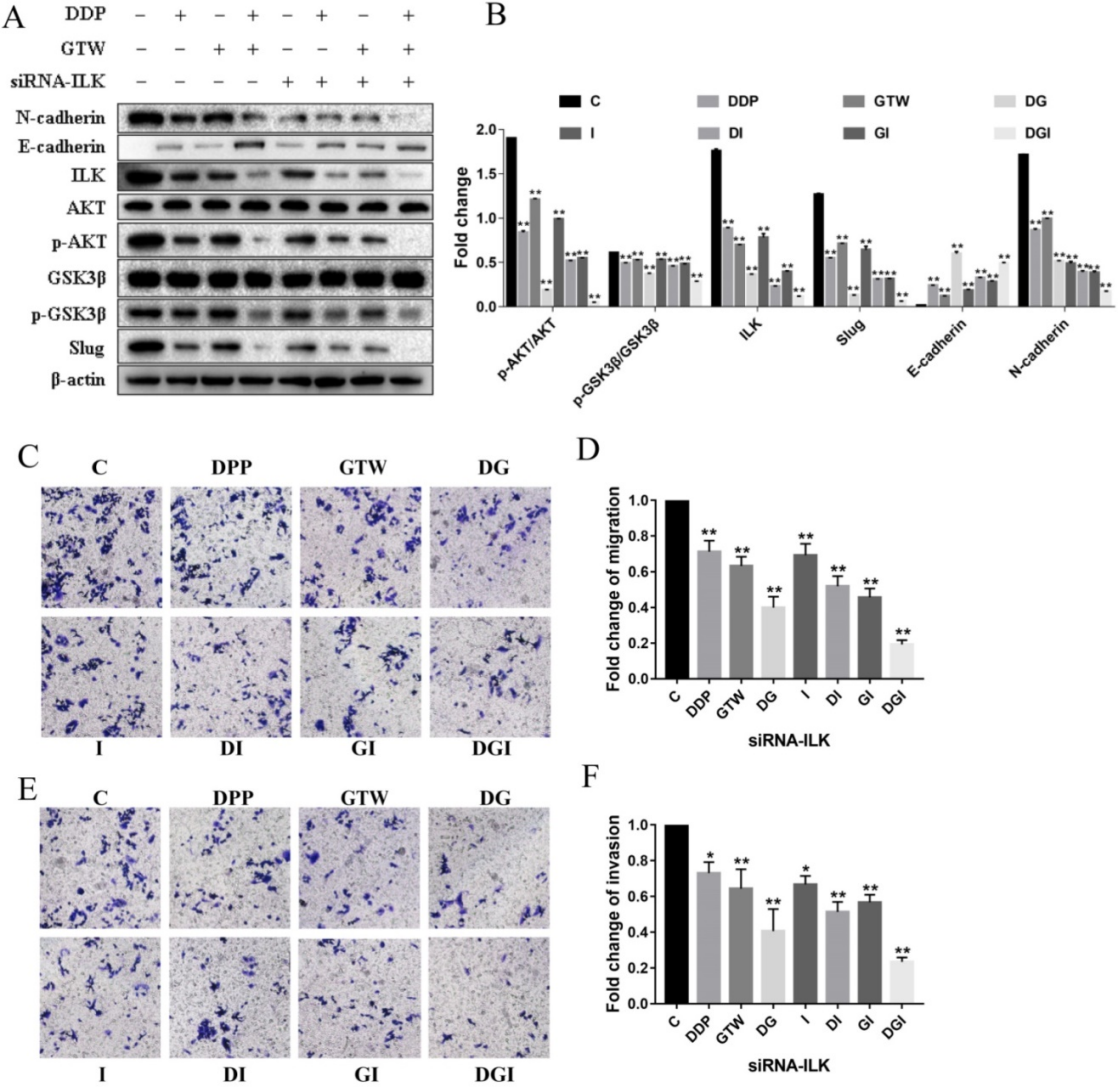
GTW repressed EMT by targeting ILK in A2780/DDP cells. (A-B) A2780/DDP cells were transfected with siRNA-ILK and treated with GTW (800 µg/mL) or DDP (10 µg/mL) for 24 hours. The expression of ILK, AKT, p-AKT, GSK-3β, p-GSK-3β, Slug, E-cadherin, and N-cadherin protein; (C-F) Transwell migration and invasion assay of A2780/DDP cells after transfection with siRNA-ILK combined with GTW, DDP or GTW+DDP treatments. *P < 0.005, **P < 0.001.

Next, we detected the levels of ILK and its downstream targets (p-AKT and p-GSK3β) in A2780/DDP cells after GTW treatment. ILK expression was significantly reduced, and the expression of p-AKT and p-GSK3β was also decreased after 24 hours of GTW or DDP incubation (Figure 3A-B). In addition, the level of Slug was also down-regulated, consistent with the results of our former research. When associating GTW with DDP, the expression of these proteins was further suppressed (*P* < 0.005). To confirm whether GTW inhibited the EMT of drug-resistant EOC cells by targeting ILK, we silenced the ILK in A2780/DPP cells by transfecting them with siRNA-ILK. After siRNA-ILK transfection, we found that the levels of ILK, p-AKT, p-GSK3β and Slug were all down-regulated in A2780/DDP cells, and the siRNA-ILK reinforced the synergistic effect of GTW and DDP combination (*P* < 0.005).

We also detected EMT related markers in A2780/DDP cells. ILK knockdown can further increase the expression of E-cadherin and suppress the level of N-cadherin in A2780/DDP cells after GTW, DDP and GTW+DDP treatment; in particular, GTW+DPP has the most obvious effect (*P* < 0.005). Furthermore, we also studied the effects of ILK silencing on cell migration and invasion followed by GTW, DDP and GTW+DDP treatment (Figure 3C-F). After siRNA-ILK transfection, the migration and invasion abilities of A2780/DDP cells were weakened, while the combination of GTW+DDP showed the most obvious inhibitory effect (*P* < 0.005). Based on the above results, we concluded that GTW inhibited EMT by targeting ILK in A2780/DDP cells.

### GTW inhibited EMT of A2780/DDP cells via the ILK/AKT signalling pathway

**Figure 4:**
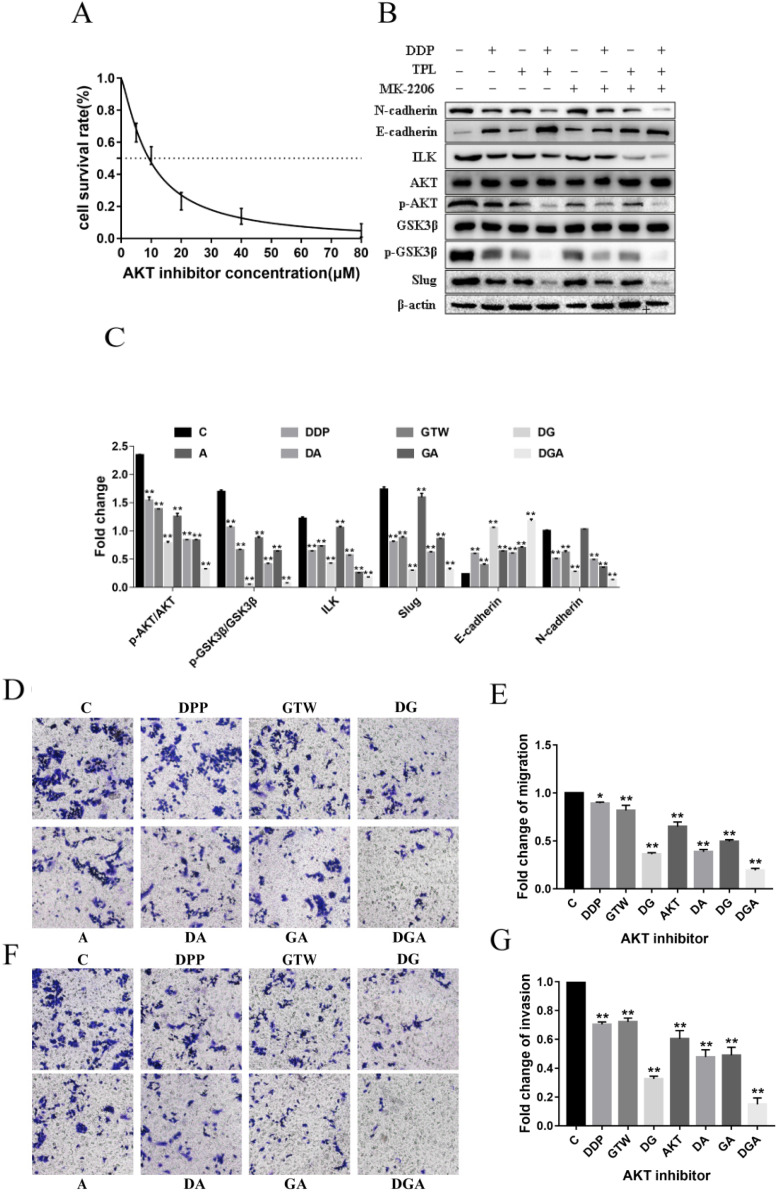
GTW suppressed the EMT of A2780/DDP cells via the ILK/AKT signalling pathway. (A) (A) A2780/DDP cells were treated with a range of concentrations of AKT inhibitor (MK2206) for 24 hours to examine cell survival; (B-C) A2780/DDP cells were treated with 10 µM MK2206 and exposed with GTW (800 µg/mL) or DDP (10 µg/mL) for 24 hours. Protein levels of ILK, AKT, p-AKT, GSK3β, p-GSK3β, Slug, E-cadherin and N-cadherin were shown; (D-G) Transwell migration and invasion assay of A2780/DDP cells after incubation with AKT inhibitor combined with GTW, DDP or GTW+DDP treatment. *P < 0.005, **P < 0.001.

To prove whether GTW inhibited EMT of A2780/DDP cells by down-regulating AKT, we incubated the cells with an AKT inhibitor (MK2206) for 6 h. As shown in Figure 4A, MK2206 inhibited the growth of A2780/DDP cells in a dose-dependent manner. MK2206 restored the GTW, DDP and GTW+DDP-induced down-regulation of p-AKT, p-GSK3β, except for ILK. Additionally, MK2206 also restored the GTW, DDP and GTW+DDP-mediated up-regulation of E-cadherin levels, and the down-regulation of N-cadherin levels in A2780/DDP cells (Figure 4B-C) (*P* < 0.005). Similarly, we conducted transwell experiments on A2780/DDP cells after MK2206 inhibitor incubation. The AKT inhibitor restored the effects of GTW, DDP and GTW DDP on migration and invasion abilities (Figure 4D-G) (*P* < 0.005). Therefore, GTW suppressed EMT of A2780/DDP via the ILK/AKT signalling pathway.

### GTW inhibits EMT of A2780/DDP cells through the ILK/AKT/GSK3β signalling pathway

**Figure 5:**
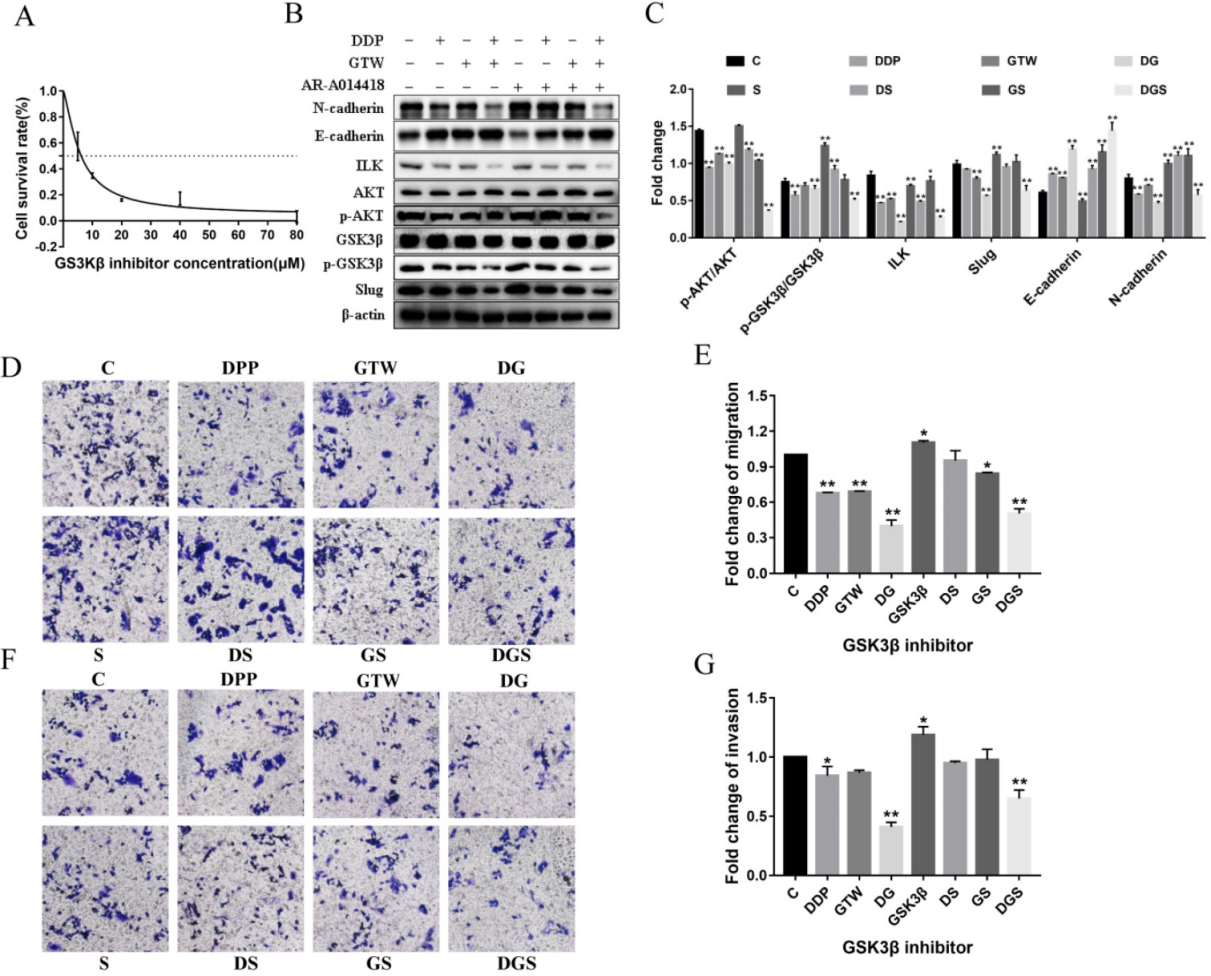
GTW suppressed the EMT of A2780/DDP cells via the ILK/AKT/GSK3β signalling pathway. (A) A2780/DDP cells were treated with a range of concentrations of GSK3β inhibitor (AR-A014418) for 24 hours to examine cell survival; (B-C) A2780/DDP cells were treated with 10 µM AR-A014418 and exposed with GTW (800 µg/mL) or DDP (10 µg/mL) for 24 hours. The expression of ILK, AKT, p-AKT, GSK3β, p-GSK3β, Slug, E-cadherin and N-cadherin protein; (D-G) Transwell migration and invasion assay of A2780/DDP cells after incubation with GSK3β inhibitor combined with GTW, DDP or GTW+DDP treatment. *P < 0.05, **P < 0.001.

To determine whether ILK acts by targeting GSK3β to suppress the EMT of A2780/DDP cells, we incubated the cells with GSK3β inhibitor (AR-A014418) at different concentrations for 6 h. As shown in Figure 5A, AR-A014418 inhibited the growth of A2780/DDP cells in a dose-dependent manner. AR-A014418 reversed the GTW, DDP and GTW+DDP-induced down-regulation of p-GSK3β and Slug expression except for ILK and AKT (*P* < 0.05). In addition, AR-A014418 also reversed the GTW, DDP and GTW+DDP-mediated up-regulation of E-cadherin levels, and the down-regulation of N-cadherin levels in A2780/DDP cells (Figure 5B-C) (*P* < 0.05). Similarly, we conducted transwell experiments on A2780/DDP cells after GSK3β inhibitor treatment (Figure 5D-G). As expected, GSK3β inhibitor reversed the effects of GTW, DDP and GTW DDP on cell migration and invasion abilities (*P* < 0.05). The above results indicated that GTW inhibited the EMT of A2780/DDP cells through the ILK/AKT/GSK3β signalling pathway.

### GTW suppressed the proliferation of human tumour cells *in vivo*

**Figure 6:**
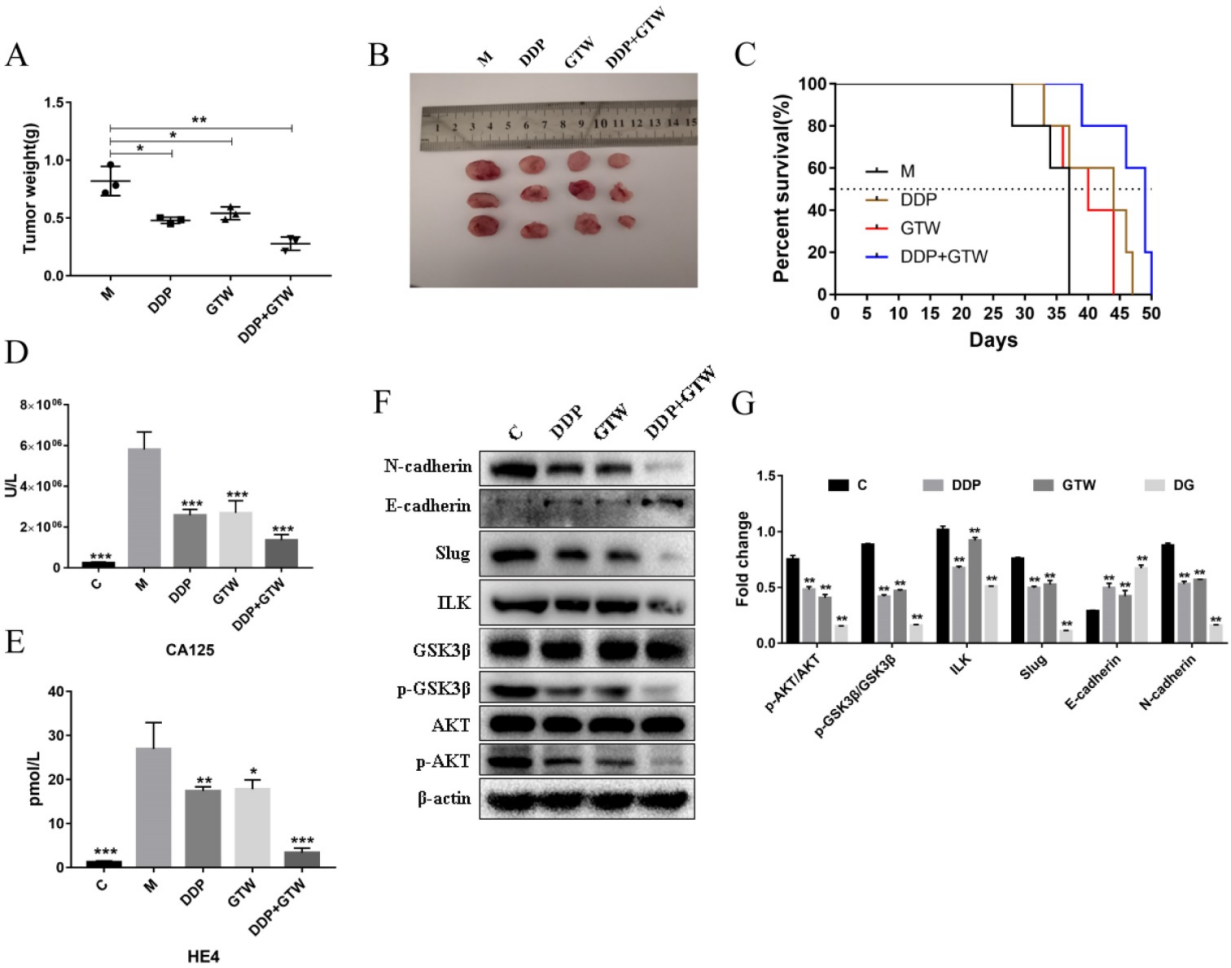
EMT repressed the proliferation, migration, and invasion abilities of A2780/DDP cells* in vivo*. (A-B) Weights (N = 3) and volumes (N = 4) of tumours in different treatment groups; (C) Kaplan-Meier survival curves of mice bearing melanoma models after the intraperitoneal administration of GTW or DDP (N = 5); (D-E) CA125 and HE4 levels in serum were measured by ELISA; (F-G) Relative expressions of ILK, AKT, p-AKT, GSK3β, p-GSK3β, Slug, E-cadherin and N-cadherin in tumour tissues. *P < 0.05, **P < 0.005, ***P < 0.001.

On the basis of the *in vitro* findings, we moved on to determine the role of GTW *in vivo*. A2780/DDP cells were injected hypodermically into the armpits of female nude mice. The anti-tumour effects were assessed after treatment with GTW alone, DDP alone and a combination of GTW+DDP. As shown in Figure 6A-B, DDP, GTW and GTW+DDP considerably delayed tumour progression, of which GTW+DDP effectively reduced the tumour volumes compared with the control group (*P* < 0.05). Also, we found that GTW, DDP and DDP+GTW can significantly prolong the survival time of mice, and DDP+GTW enhanced the survival rate up to day 50 (Figure 6C).

Furthermore, we studied the expression of serum tumour markers CA125 and HE4. As shown in Figure 6D-E, both GTW and DDP reduced the levels of CA125 and HE4 in nude mice serum, while DDP+GTW showed a stronger inhibitory effect (*P* < 0.005). Therefore, we believed that GTW combined with DDP could improve the prognosis of mice by reducing the serum levels of CA125 and HE4.

### GTW inhibited the EMT of tumour cells through the ILK/AKT/GSK3β/Slug pathway *in vivo*

The tumours removed from nude mice were subjected to western blot assays. It was found that both GTW and DDP up-regulated the level of E-cadherin and down-regulated the levels of N-cadherin, ILK, p-AKT p-GSK3β, and Slug. DDP+GTW had a more obvious regulatory effect on the above related-protein expressions in tumour tissues (Figure 6F-G) (*P* < 0.05). The above data showed that GTW suppressed the EMT of A2780/DDP cells by targeting ILK/AKT/GSK3β/Slug* in vivo*.

## Discussion

Among cancers in women, EOC has the highest fatality-to-incidence ratio. Most patients are diagnosed at an advanced stage with large-scale metastasis and development of chemo-resistant properties 32. EMT is a process whereby cancer cells achieved migratory and invasive properties as a result of alterations in cell-cell adhesion, cell polarity and cell-extracellular matrix interaction 33. Therefore, it is related to cancer metastasis and the acquisition of chemotherapy resistance properties of other cancers such as colorectal cancer 34, lung cancer 35, and pancreatic cancer 36. Previous studies showed that TPL inhibited the migration and invasion abilities of drug-resistant EOC cells *in vitro* and *in vivo* 29-31. However, whether GTW has the ability to modulate EMT and drug resistance of DDP-resistant EOC cells and the potential mechanisms are still not fully understood.

The absence or decreased expression of E-cadherin is regarded as a major characteristic of EMT that is a consequence of the actions of a number of TFs (Snail, Slug, Twist, ZEB1/2 and SIP1) that target the E-box sequences within the promoter region of E-cadherin 37-39. Slug is closely related to tumour metastasis of ovarian cancer 40. In this study, we found that the proliferation, migration and invasion of A2780/DDP cells were decreased in a dose-dependent manner after GTW treatment (Figure 1). GTW, especially GTW+DDP, could up-regulated E-cadherin expression and mediated N-cadherin down-regulation. We found that GTW suppressed the migration and invasion abilities of A2780/DDP cells and strengthened cell-sensitivity to DDP by inhibiting EMT (Figure 2). Slug was also suppressed after GTW incubation. Additionally, Slug silence suppressed EMT, which was verified by molecular and functional experiments. Based on this, we firmly believed that GTW inhibited EMT of A2780/DDP cells by targeting Slug (Figure 2).

Accumulating genetic and cancer biology evidence demonstrate that the PI3K/AKT signalling pathway is a central mechanism controlling EMT features, for its definite effects on cancer cell growth and survival 16, 41. As the upstream regulatory molecule of AKT, ILK is a key factor that regulates the interaction between extracellular matrix and cells by phosphorylating of its downstream substrates AKT and GSK3β 42. ILK has significant effects on the development and progression of human carcinoma 20, 43, 44, including promoting the metastatic behaviour of EOC cells 45. It has been reported that ILK facilitates cancer cell migration, invasion and drug-resistance by regulating EMT processes 46. What is more, emodin restrained EMT through the ILK/AKT/mTOR signalling pathway in breast cancer 47. However, whether GTW can suppress ILK level in DDP-resistant EOC cells is unclear. In our study, we observed that GTW, especially GTW+DDP could significantly inhibit the expression of ILK in A2780/DDP cells. In order to clarify how ILK regulate EMT, we transfected A2780/DDP cells with siRNA-ILK to knock down the ILK expression. GTW, especially GTW+DDP, strengthened the influence of siRNA-ILK on the expression of E-cadherin and N-cadherin. In addition, siRNA-ILK inhibited the migration and invasion abilities of A2780/DDP cells. The existing research results showed that GTW weakened the migration and invasion abilities of A2780/DDP cells and strengthened cell-sensitivity to DDP by inhibiting EMT via targeting ILK (Figure 3).

ILK acts as a vital regulator in the PI3K/AKT pathway to phosphorylate its downstream targets such as AKT and GSK3β to regulate their activities 44. ILK interacts with AKT and stimulates phosphorylation of AKT, leading to its activation. ILK can also direct and/or indirect stimulate phosphorylation of GSK3β. In addition, AKT participates in regulating the wingless/integrated (Wnt)/β-catenin signalling pathway by phosphorylating GSK3β, leading to the inactivation of GSK3β 48, 49. In hepatocellular carcinoma (HCC), transfection with an ILK expression vector was able to recover the decreased expression of its downstream genes AKT and GSK3β phosphorylation, and affected cell proliferation and apoptosis 44. In oral squamous cell carcinoma cells (OSCC), the knockdown of ILK level inhibited EMT by suppressing the phosphorylation of downstream signalling targets AKT and GSK3β both *in vitro* and *in vivo* 50. In the present study, we confirmed that GTW, especially GTW+DDP, inhibited EMT by targeting ILK and the expression of p-AKT and p-GSK3β was down-regulated, consistent with ILK (Figure 3). Moreover, siRNA-ILK significantly decreased the levels of ILK, p-AKT, p-GSK3β, and Slug in A2780/DDP cells, which were reinforced by GTW, especially GTW+DDP (Figure 3). The AKT inhibitor reversed the GTW and GTW+DDP-induced effects of E-cadherin, N-cadherin, p-AKT, p-GSK3β, and Slug, except ILK (Figure 4). Similarly, in addition to ILK and p-AKT, the GSK3β inhibitor restored the effects of E-cadherin, N-cadherin, p-GSK3β and Slug induced by GTW and GTW+DDP (Figure 5). In terms of xenografts in nude mice, GTW, especially GTW+DDP, could decelerate tumour progression, prolong the survival rate of mice and down-regulate tumour-related protein expression (Figure 6). To sum up, our data confirmed that GTW inhibited EMT related metastasis and chemo-resistance (DDP-resistance) both *in vitro* and* in vivo* through the ILK/AKT/GSK3β/Slug pathway.

Cancer antigen 125 (CA125) is a commonly and initially used tumour biomarker that is enhanced in 80% of females with advanced-stage ovarian cancer 51, and higher level of CA125 usually indicates poor treatment outcomes and prognosis 52. Human epididymis protein 4 (HE4) is considered a new biomarker for diagnosing ovarian cancer either at the early or late stages. In addition, HE4 levels also had good sensitivity in ovarian cancer patients when monitoring the prognosis condition postoperatively 53. Overall, the combination of the two markers is more reliable and will increase the predictive value 54, 55. In our research, we showed that GTW, especially GTW+DDP could significantly reduce the levels of CA125 and HE4 in the serum of nude mice (Figure 6). The above results indicated that GTW can improve the prognosis of drug-resistant EOC and strengthened cancer cell-sensitivity to DDP.

## Conclusion

Overall, our study indicated that GTW can inhibit the proliferation, migration and invasion abilities as well as intensify sensitivity to DDP of A2780/DDP cells by suppressing EMT *in vitro* and *in vivo*. Furthermore, we confirmed that the role of GTW in EMT was regulated via the ILK/AKT/GSK3β/Slug signalling pathway. All of these results indicate that GTW is used as an adjuvant therapy for drug-resistant EOC in clinic. However, the lack of normal and other cancer cells as internal and external controls makes it a limitation in the present study.
